# The Quest for Materials-Based Hydrogels with Antimicrobial and Antiviral Potentialities

**DOI:** 10.2174/1874357901812010069

**Published:** 2018-08-31

**Authors:** Hafiz M. N. Iqbal

**Affiliations:** Tecnologico de Monterrey, School of Engineering and Sciences, Campus Monterrey, Ave. Eugenio Garza Sada 2501, Monterrey, N.L., CP 64849, Mexico

**Keywords:** Biomaterials, Hydrogels, Biomedical applications, Antimicrobial, Antiviral, Polymeric network

## Abstract

In recent years, the Antimicrobial Resistance (AMR) or Multidrug Resistance (MDR) and viral infections have become serious health issues, globally. Finally, after decades of negligence, the AMR/MDR and viral infection issues have now captured a worldwide attention of the global leaders, public health community, legalization authorities, academia, research-based organizations, and medicinal sector of the modern world, alike. Aiming to resolve these issues, various methodological approaches have been exploited, in the past several years. Among them, biomaterials-based therapeutic hydrogels are of supreme interests for an enhanced and efficient delivery in the current biomedical sector. Depending on the regulatory authorities and practices, the antibiotics consumption was expedited than ever before driven by rising and increasing access, across the globe. Though the emergence of AMR/MDR in microorganisms and emergence/reemergence of viral infections are considered as a natural phenomenon, however, these concerning issues have been driven by those mentioned above faulty human behavior. In this context, many scientists, around the globe, are working at wider spectrum to resolve this problematic issue, efficiently. A proper understanding of biological mechanisms is essential to combat this global threat to the living beings. In this review, an effort has been made to highlight the potent features of materials based hydrogels possessing antimicrobial and antiviral potentialities. The information is also given on the potential research activities, and possible mechanisms of actions of hydrogels are discussed with a closeup look at the future recommendations.

## 1 INTRODUCTION

Materials-based hydrogels are a three-dimensional cross-linked polymeric network which can be multifunctional in nature and able to respond to the external stimuli *e.g.* environmental pH, temperature, light, electric field, *etc*. Most often, the biomaterials *e.g.* chitosan, *etc*. based hydrogels possess excellent biocompatibility features, thus are broadly practiced in various sectors of pharmaceutical, cosmeceutical and biomedical engineering [1-4]. Also, owing to the water holding capacity, hydrogels have an extraordinary capability to swell and/or de-swell depending on the environmental conditions accordingly. The available hydrophilic groups or functional domains *i.e.* carboxyl, amino, and/or hydroxyl groups in the polymeric network play a major role in the water holding capacity of the hydrogels [5]. The hydrophilic groups or functional domains and water holding feature of a hydrogel are directly proportional to each other. Higher the hydrophilic groups or functional domains, higher will be the swelling or water holding capacity. The swelling behavior can be controlled by mimicking the cross-linking density of a polymeric network. During water exposure, water penetrates into the polymeric networks causing swelling, thus responsible for giving hydrogel its shape. Likewise, a living tissue, a hydrogel in its complete swollen state has some unique characteristics such as soft and rubbery consistency along with a low interfacial tension with water or biological fluids [6-8].

In recent years, the quest for materials-based hydrogels with potent functionalities including antimicrobial potentialities has revitalized the field of biomaterials. Fig. (**1**) illustrates a simplified synthesis of the materials-based hydrogel in which a material of interest is polymerized in the presence of a functional cross-linking agent. Whereas, a bio-responsive behavior of control drug (antimicrobial/antiviral) release subject to pH and temperature change is shown in Fig. (**2**) [3]. With ever-increasing scientific awareness, novel approaches and technologies in the hydrogel design have been introduced that offer with significantly improved mechanical properties, super porous, and comb-type grafted hydrogels [9-14]. Other examples of hydrogel biomaterials with a smart future include, self-assembled hydrogels with tunable protein domains, stimuli (external/internal) responsive hydrogels, and genetically engineered triblock copolymers based hydrogels [15-18].

The present review work focuses on the development of novel types of materials-based hydrogels with antimicrobial and antiviral potentialities for various biomedical sectors of the modern world. Herein, this review is not presenting the whole literature about biomaterials as it is a big field with huge information. Thus, the focus is only given to the hydrogels perspective. The AMR/MDR resistance issues along with viral infections have been highlighted along with the possible action mechanisms of the hydrogels are briefly discussed with a closeup look at the future recommendations. Towards the end, outstanding issues posing questions mark and needs to be addressed are presented that can pave the way for future studies.

### 2 BIOMATERIALS - A BIOMIMETIC APPROACH

The development of new types of novel, effective and highly reliable materials-based hydrogels for multipurpose applications is essential and a core demand to tackle many human health related diseases. Owing to the unique chemical structure, bioactivity, non-toxicity, biocompatibility, biodegradability, recyclability, *etc*. bio-based materials possesses several complementary functionalities that position them well in the materials sector of the modern world. Many bio-based materials including chitin, chitosan, bacterial cellulose, alginate, and keratin, among others have been fully characterized and well organized/developed into value-added structures [19-33]. Thus, provide a proper route to emulate bio-systems - a biomimetic approach to eliminate the concerns like unfavorable immune responses, disease transmission, effective wound healing, control delivery and regeneration potentialities [34, 35].

### 3 AMR/MDR RESISTANCE ISSUES

With ever increasing scientific knowledge and social awareness, now the people are more concern about the AMR/MDR issues. The increasing challenge to health care attributable to the AMR/MDR, therefore, AMR has become a worldwide concern, in recent years [36-41]. Broadly speaking, AMR is defined as a temporary and/or permanent capability of a microbial strain and its progeny to resist and/or stay viable and multiply against the medication previously used to treat them. Owing to this notable resistivity and non-susceptibility, microbes have been classified as resistant strains to the concentration of an antimicrobial agent used in practice [42]. The AMR/MDR is a growing problem at the global level. Developing a range of strategies to reduce reliance on antimicrobials will be a key challenge for the future [43]. Owing to the antibiotic-resistant, infections now account for 25,000 deaths in Europe alone (European Centre for Disease Prevention and Control), and about 23,000 deaths and over 2 million illnesses in the US (Centers for Disease Control and Prevention), annually. In September 2014, the US President “Obama” signed an Executive Order instructing key health agencies to take action to combat the rise of antibiotic-resistant bacteria [44]. Owing to the emerging or re-emerging infectious diseases caused by various microorganisms, much attention is now being focused towards alternative approaches to control and/or limit such deadly infections. In this context, novel materials with antimicrobial activities are attracting the considerable attention of both academia and industry, especially in the biomedical, and other health-related sectors of the modern world [19, 23-25, 27]. Because of the growing consciousness and demands of legislative authorities, the manufacture, to reduce bacterial population in healthcare facilities and possibly to cut pathogenic infections, development of novel anti-microbial active materials which are biocompatible and biodegradable are considered to be a potential solution to such a problematic issue.

Among the potential causes, below are some possible explanations for an increased AMR/MDR:

1. The genetic transformation from strain to strain.
2. Biofilm matrix forming potential of several strains.
3. Efflux pumps and other outer membrane structural variations.
4. Enzyme-mediated resistance against, in practice, antimicrobials.
5. Enhanced level of metabolic activity within the biofilm structure.
6. Lower/no perfusion of antimicrobial agents through the biofilm matrix.
7. Adaptability and interaction between antimicrobial agents and biofilm matrix.
8. Excessive/useless consumption of in practice antimicrobials in a random order.
9. Genetic variation and adaptability against the excessive antimicrobials exposure.

### 4 CLASSIFICATION OF HYDROGELS

**Fig. (3):**
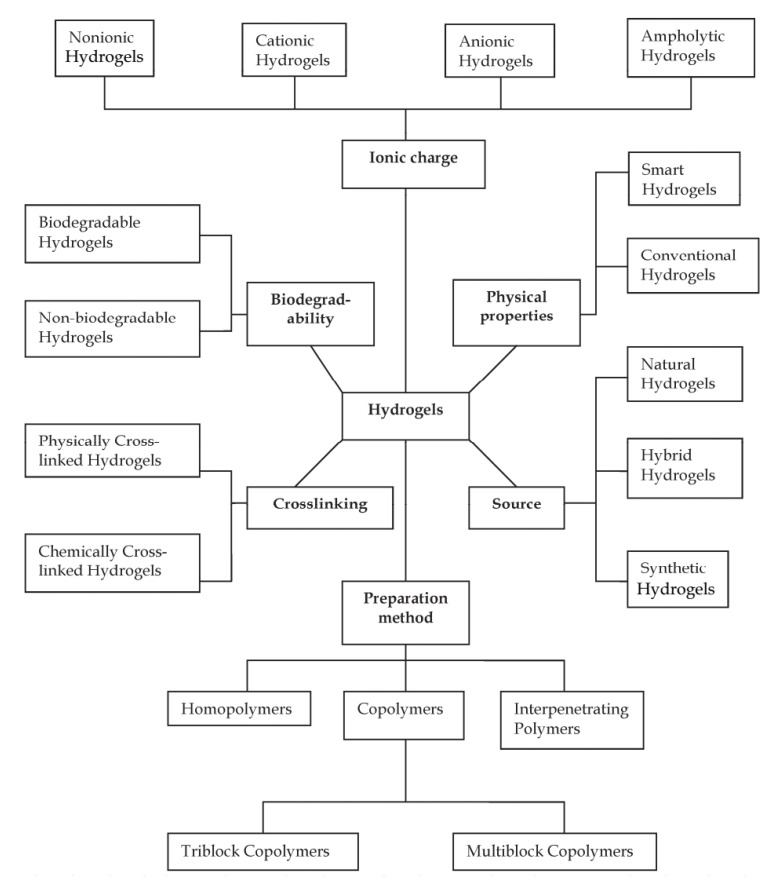
Classification of Hydrogels (Reproduced from Ref [48], an open access article distributed under the terms of the creative commons attribution License).

Based on the literature data, hydrogels have been classified in several ways depending on the preparatory techniques, materials sources, ionic charges, stimuli (external or internal) responsiveness, crosslinking nature and biodegradability characteristics, *etc*. Though each method has its specificity and uniqueness, however, among all of the ways mentioned above, one of the important classifications is based on their crosslinking nature (Fig. **3**) [44-48]. The crosslinking nature either chemical or physical plays a critical role in the network stability of hydrogels in their swollen state [48]. A detailed classification of hydrogels along with the preparation and potential applications of each sub-classified type are reviewed elsewhere [1, 48-55], thus not the focus of this review.

### 5 TECHNOLOGICAL FEATURES OF HYDROGELS

Owing to the diversity, hydrogels offer numerous technical and functional features. For example:

1. Overall cost-effective ratio
2. Minimal residual monomer
3. Maximal absorption capacity
4. Colorlessness, odourlessness
5. Swelling and de-swelling property
6. Tunable particle size and porosity
7. Stability, compatibility, and biodegradability

The above-listed features are just considerable examples and are tunable depending on the materials used and requisite application.

### 6 HYDROGELS WITH ANTIMICROBIAL POTENTIALITIES

Hydrogels with antimicrobial potentialities are envisioned to be a fundamental weapon to combat AMR/MDR infections. Recently, the emergence of AMR/MDR microbial strains has created an innumerable challenge within the healthcare arena. Much sadly, current problems associated with the AMR/MDR microbial strains outspread far beyond gram-positive bacteria such as MRSA [56]. In this context, there is a demanding need to engineer antimicrobial active hydrogels using above mentioned materials. As discussed above, the antimicrobial is a requisite property, and the material *e.g.* hydrogel should possess the following characteristics [57].

1. Eco-friendlier processing conditions
2. Highly stable with a long-term shelf life
3. Does not generate toxic products/byproduct, while in use
4. High level of biocidal against a wider spectrum of pathogenic microbes

Among many potent materials, chitosan as a natural polyaminosaccharide possesses many of the above-attributed features. Thomas *et al*. [58] reported a facile *in-situ* procedure to fabricate hydrogel–silver nanocomposites and investigation of their antimicrobial activity. The process involves the formation of silver nanoparticles within swollen poly (acrylamide-*co*-acrylic acid) hydrogels. In the same study, the authors have demonstrated excellent antibacterial effects of the developed hydrogel–silver nanocomposites against *Escherichia coli*. Antibacterial activity of a chitosan–γ-poly (glutamic acid) polyelectrolyte hydrogel has been reported by Tsao and co-workers [59]. The developed hydrogel was comprised on chitosan as the cationic polyelectrolyte and γ-poly(glutamic acid) (γ-PGA) as the anionic polyelectrolyte. The chitosan–γ-PGA hydrogels exhibited antibacterial activity against *Escherichia coli* and *Staphylococcus aureus*. Later, the same group of authors have evaluated the chitosan/γ-poly (glutamic acid) polyelectrolyte complex hydrogels and proved useful for wound-healing capabilities [60]. Though chitosan is well known to exhibit considerable antibacterial activity against different microbial strains, however, there are some limitations regarding its molecular weight. Based on the literature data, the chitosan with low molecular weight has higher/stronger biocidal/antibacterial activity against *Escherichia coli* than that of the chitosan with higher molecular weight [61]. Likewise, in an earlier study, No *et al*. [62] reported antibacterial activities of six chitosans and six chitosan oligomers with different molecular weights against four gram-negative strains *i.e.* *Escherichia coli*, *Pseudomonas fluorescens*, *Salmonella typhimurium*, and *Vibrio parahaemolyticus* and seven gram-positive strains *i.e.* *Listeria monocytogenes*, *Bacillus megaterium*, *B. cereus*, *Staphylococcus aureus*, *Lactobacillus plantarum*, *L. Brevis*, and *L. bulgaricus*. The development of a novel hydrogel based on dimethyl alkyl ammonium chitosan (with various degrees of quaternization)-graft-poly(ethylene glycol) methacrylate (qC-g-EM) and poly(ethylene glycol) diacrylate with potent antibacterial activity have been reported [63]. The reported hydrogel has excellent antimicrobial efficacy against *Pseudomonas aeruginosa*, *Escherichia coli*, *Staphylococcus aureus and Fusarium solani*. In the same study, authors reported that increasing the alkyl chain length of the quaternizing agent from Trimethylammonium (TM) to dimethyldecylammonium (DMD) led to greater efficacy against Gram-positive strain *i.e.* *S. aureus* but not the Gram-negative strain *i.e.* *E. coli* and *P. aeruginosa*.

### 7 ANTIMICROBIAL MECHANISM OF HYDROGELS

The antimicrobial mechanism of hydrogels from the materials with intrinsic antimicrobial activity is not fully understood. Various authors have proposed and justified antimicrobial mechanism of materials or materials based hydrogels and constructs [19, 23-25, 27, 38, 63-66]. Most of the reported antimicrobial mechanisms are based on the bacterial cell membrane lysis subject to the available reactive functional groups and routes. The anionic bacterial cells are attracted to the cationic materials or materials based hydrogels through electrostatic interaction, leading to subsequent cell wall disruption, membrane lysis, cell leakage that ultimately leads to cell death. In summary, the following points play major/critical role in the antimicrobial activity of materials or materials based hydrogels.

1. Charge (cationic) surface ratio
2. Amphiphilicity of the materials
3. The available alkyl chain length
4. Availability of reactive functional groups
5. Overall pore size of the materials final product
6. Permeability features of the target (microbial) membrane

### 8 HYDROGELS WITH ANTIVIRAL POTENTIALITIES

In practice antiviral therapeutics *e.g.* oseltamivir and zanamivir, *etc*. are facing increasing problems with resistance development. Oseltamivir is a selective antiviral prodrug which is used to tackle influenza virus. Whereas, zanamivir is an inhibitor of neuraminidase used in the treatment of common flu and in the prophylaxis of virus A and B. Engineering efficient antiviral drugs with potent activities against a wider spectrum of viral pathogens is difficult because viruses use the host's cells to replicate. Therefore, researchers, around the globe, are working to extend the range of antivirals to other families of pathogens. Owing to the ever-increasing drug resistance, there is an urgent need to develop novel formulations in a range of contexts to tackle various viral infections. Furthermore, the constantly changing genetic makeup of viruses may alter or induce the viral resistance against several in-practice treatment strategies [67]. Spontaneous or intermittent mechanisms are mainly responsible for viral resistant throughout the antiviral treatment. In an earlier study, Herlocher *et al*. [68] isolated three type A influenza viruses, each of which has a distinct neuraminidase-gene mutation and is resistant to the neuraminidase inhibitor oseltamivir. Likewise, immunocompromised patients, who received oseltamivir for “post-exposure prophylaxis” are also at higher risk of resistance [69].

In this context, biomaterials-based hydrogels with antimicrobial “non-drugs” *e.g.* nanoparticles find widespread biomedical applications [70]. Such biomaterials-based hydrogels systems with incorporated compounds offer additional advantages *e.g.* low or no toxicity, responsive behavior, favorable tissue integration, targeted and control drug release potentialities, and controlled degradation rate. With these added values, considerable research efforts have been made to incorporate antiviral drugs into hydrogels. For example, Thorgeirsdottir *et al*. [71] reported that for monocaprin solutions containing 5% propylene glycol, the antiviral activity is greatly reduced by 5% polysorbate 20. In the same study, authors have also revealed that solutions containing 7.5% propylene glycol and polysorbate 20 (0.75 to 1.5% concentration) were found to have antiviral activities comparable to that of pure monocaprin [71]. Considering antiviral agents, monocaprin is one among the highly efficient monoglycerides. It has also been shown to be effective against the enveloped viruses *e.g.* vesicular stomatitis virus, Herpes Simplex Virus (HSV), visna virus and human immunodeficiency virus *in-vitro* [72, 73]. Similarly, hydrophilic gels containing monocaprin in a concentration of 20 mM have shown more than 100,000-fold inactivation of HSV-2 and HSV-1 [72, 73]. In another study, Rokhade *et al*. [74] developed semi-interpenetrating polymer network microspheres of acrylamide-grafted dextran and chitosan-based hydrogels for controlled release of antiviral drug acyclovir. Chiappetta *et al*. [75, 76] incorporated antiviral drug efavirenz into PEO/PPO block copolymer hydrogels for pediatric anti-HIV pharmacotherapy with significantly higher oral bioavailability.

### 9 CONCLUDING REMARKS AND FUTURE CONSIDERATIONS

In conclusion, this review highlights the potent features of materials based hydrogels possessing antimicrobial and antiviral potentialities. Herein, the work was aimed to critically overview the literature to establish an infective capacity of the naturally occurring materials or materials based hydrogels using a range of microbiological techniques against viral infections and various gram-positive and gram-negative pathogenic microbial strains including antibiotic-resistant forms. Through cautious strategy, the tunable materials or materials based hydrogels with multi-functionalities can be engineered to achieve optimal infective capability and therefore enhanced AMR/MDR control. Moreover, a novel type of potent materials could be designed for the management and skin regeneration/repair from injury, particularly burns and ulcers, where the risk of bacterial infection is high. Material structure and performance integrity need to be accessed using a range of analytical and imaging techniques.

Aiming to treat or tackle microbial and/or viral infections, a precise and control delivery of engineered constructs with diverse bioactivities is critical. The biocidal agents must be released from the gels to the target site to kill/eradicate the infectious agents in a safer and sustained manner. Such careful practices are essential to treat infections effectively to prevent biofilm formation that can play a crucial role in the development of resistant strains. Against, AMR/MDR strains, a strong synergistic effect can be achieved by engineering hydrogels using materials with intrinsic antimicrobial potential along with the impregnation of conventional antimicrobial agents.

### 10 OUTSTANDING QUESTIONS AND RESEARCH GAPS

Despite the huge research and plethora of reported literature on materials-based constructs including hydrogels, there are still outstanding issues posing questions mark and needs to address.

1. Is it a cost-effective process to develop gels using multi-materials co-supported with other conventional antimicrobial agents?
2. What could justify the development of gels which are active only against a certain number of strains instead of the wider-spectrum ones?
3. What is the maximum alkyl chain length for the generation of a bactericidal efficacy at an optimal level?
4. How can a robust methodology be designed to standardize the information and satisfy regulatory concerns?
5. What are the ultimate and long-end consequences of the increased use of conventional antimicrobial agents?
6. What are the health and environmental impacts of the heavy consumption of natural materials?

## Figures and Tables

**Fig. (1):**
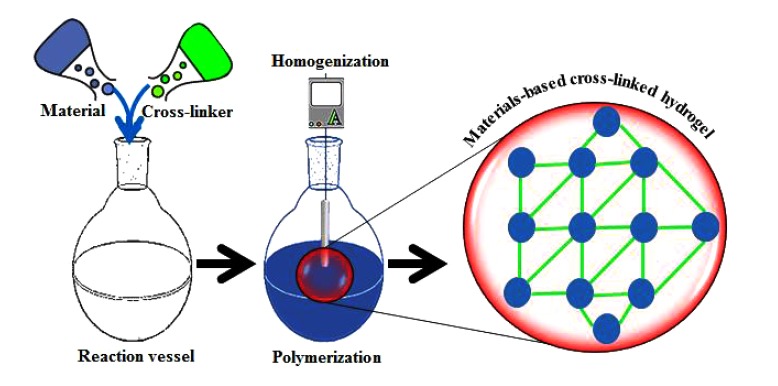
Schematic representation of materials-based hydrogel preparation in the presence of the functional cross-linking agent.

**Fig. (2):**
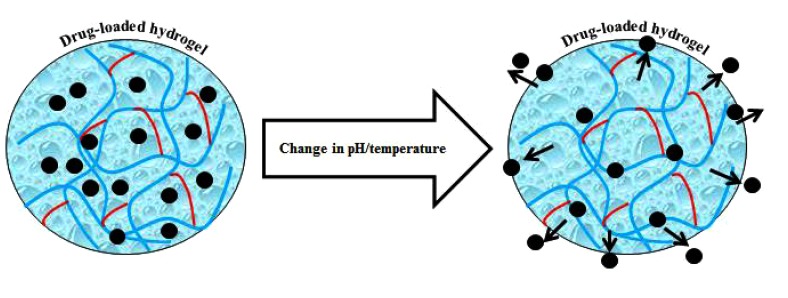
The bio-responsive behavior of control drug release subject to pH and temperature change. The drug molecules could be antimicrobial or antiviral depending on the requirement (Reproduced from Ref [3], an open-access article distributed under the terms of the creative commons attribution License).
